# Docosahexaenoic Acid Is Naturally Concentrated at the *sn*-2 Position in Triacylglycerols of the Australian Thraustochytrid *Aurantiochytrium* sp. Strain TC 20

**DOI:** 10.3390/md19070382

**Published:** 2021-07-01

**Authors:** Kim Jye Lee-Chang, Matthew C. Taylor, Guy Drummond, Roger J. Mulder, Maged Peter Mansour, Mina Brock, Peter D. Nichols

**Affiliations:** 1 CSIRO Oceans & Atmosphere, Hobart, TAS 7000, Australia; Mina.Brock@csiro.au (M.B.); Peter.Nichols@csiro.au (P.D.N.); 2 CSIRO Land & Water, Canberra, ACT 2601, Australia; M.Taylor@csiro.au; 3 Pharmamark Nutrition Pty Ltd., Eight Mile Plains, QLD 4113, Australia; Guyd@pharmamark.com.au; 4 CSIRO Manufacturing, Clayton, VIC 3169, Australia; Roger.Mulder@csiro.au; 5 CSIRO Agriculture & Food, Werribee, VIC 3030, Australia; Peter.Mansour@csiro.au; 6 Institute Marine and Antarctic Studies, University of Tasmania, Hobart, TAS 7005, Australia

**Keywords:** thraustochytrids, regiospecificity, triacylglycerols, polyunsaturated fatty acids

## Abstract

The Labyrinthulomycetes or Labyrinthulea are a class of protists that produce a network of filaments that enable the cells to glide along and absorb nutrients. One of the main two Labyrinthulea groups is the thraustochytrids, which are becoming an increasingly recognised and commercially used alternate source of long-chain (LC, ≥C_20_) omega-3 containing oils. This study demonstrates, to our knowledge for the first time, the regiospecificity of the triacylglycerol (TAG) fraction derived from Australian thraustochytrid *Aurantiochytrium* sp. strain TC 20 obtained using ^13^C nuclear magnetic resonance spectroscopy (^13^C NMR) analysis. The DHA present in the TC 20 TAG fraction was determined to be concentrated in the *sn*-2 position, with TAG (16:0/22:6/16:0) identified as the main species present. The *sn*-2 preference is similar to that found in salmon and tuna oil, and differs to seal oil containing largely sn-1,3 LC-PUFA. A higher concentration of sn-2 DHA occurred in the thraustochytrid TC 20 oil compared to that of tuna oil.

## 1. Introduction

Dietary consumption of omega-3 long-chain (≥C_20_) polyunsaturated fatty acids (LC-PUFA, also termed LC omega-3), in particular docosahexaenoic acid (DHA, 22:6ω3), has many benefits in human health. Omega-3 fatty acids are essential in the diet as they are not synthesised. DHA is enriched in the brain at up to 15% of the total fatty acid (FA) pool and has been shown to be essential for neuronal and retinal development and function [1]. Studies have shown that the consumption of LC omega-3 oil also helps prevent heart disease, neural disorders, arthritis, asthma, and skin diseases in humans [2,3].

Whilst omega-3 LC-PUFA are found in fish oils, including from commercially available nutritional supplements derived from fish oils, marine microbes including bacteria, fungi, algae, and plankton are actually the fundamental source of these key and essential FA in the marine ecosystem. They constitute a major food source for marine organisms at the base of the food web. For example, species of the genera *Thraustochytrium*, *Nannochloropsis*, *Attheya*, *Pseudonitzschia*, and *Rhodomonas* possess the ability to produce a number of LC omega-3 containing oils, especially DHA and eicosapentaenoic acid (EPA, 20:5ω3). This has led to a growing interest in the use of marine protist class thraustochytrid-derived oils for human nutrition and aquaculture feed, with nutritional and functional benefits [4,5]. Our previous studies have shown the high triacylglycerol (TAG) content in strain TC 20, containing high proportions of omega-3 LC-PUFA, in particular DHA [6]. 

The dietary sources of DHA can affect its uptake and translocation. DHA can be transported across the blood brain barrier from serum pools of either lysophosphatidic acid or non-esterified fatty acid [7]. Clinical trials on humans and animal testing have demonstrated that the regiospecificity of fats can affect the nutritional value of the lipids. This is due to the process of TAG hydrolysis being catalysed by gut lipase enzymes, which work specifically at the *sn*-1, 3 position [8]. As a result, fatty acids occurring in the *sn*-2 position will be retained on the TAG backbone, and are more readily absorbed in the process of metabolising lipids in the body [8,9]. Numerous studies have shown that the dietary uptake of DHA is more efficient if the DHA is located at the *sn*-2 position of the glycerol backbone [9,10]. In hamsters, it has been shown that DHA on the *sn*-2 position of oils is more efficiently translocated to other tissues including the brain, liver, and serum than DHA at the *sn*-1,3 position or ethyl ester DHA [11]. Similar results have also been reported for other FA such as palmitic acid (PA, 16:0) which is predominantly present in the *sn*-2 position (over 70%) in human milk, while most of the 16:0 (75–97%) in infant formulas was located in the outer positions of the triacylglycerols, at *sn*-1,3 [12,13]. It has also been demonstrated that dietary *sn*-2 PA increases PA in the *sn*-2 position of TAG in plasma obtained from term infants [13]. 

In addition, the effect of a reduction in cholesterol and TAG levels in the blood is related to the distribution and regiospecific position of EPA and DHA on TAG [9]. With the above background, an understanding of TAG regiospecificity of these thraustochytrid-derived LC omega-3 oils is clearly important, because these key omega-3 LC-PUFA will accumulate in food chains, eventually pass through the food web, and make their way into animals that we consume. In addition, such information may be important for consumer awareness and enhancing our understanding of potential health implications as there are an increasing range of traditional and alternative sources of omega-3 oils available on the market.

This paper describes, to our knowledge, for the first time the positional distribution of DHA occurring on the glycerol backbone of triacylglycerol fractionated from Australian Thraustochytrid *Aurantiochytrium* sp. strain TC 20. The results may have future relevance towards better understanding the potential nutritional and functional benefits for specific food and nutraceutical applications.

## 2. Results and Discussion

**Figure 1:**
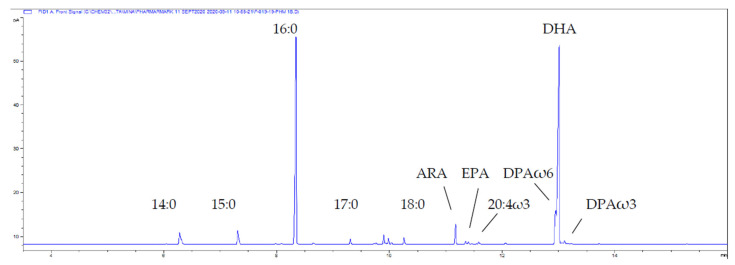
Partial gas chromatogram of the total lipid-derived FA (as FAME) of the thraustochytrid *Aurantiochytrium* sp. strain TC 20. The shoulder on the front of DHA is DPAω6. Abbreviations: Arachidonic acid ARA, 20:4ω6; EPA, 20:5ω3; Docosapentaenoic acid DPAω3, 22:5ω3; DPAω6, 22:5 ω6; DHA, 22:6ω3.

**Figure 2:**
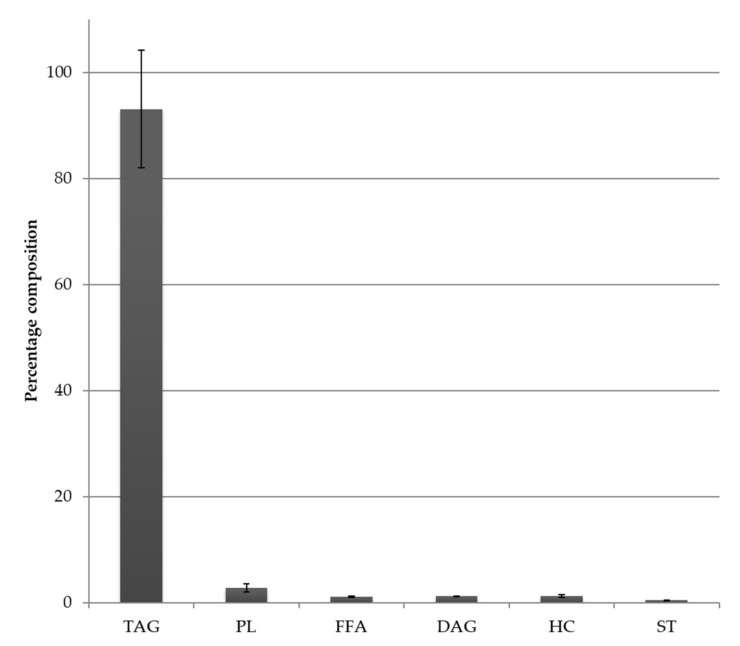
Lipid class composition (as a percent of total lipid) of the total lipid of the thraustochytrid *Aurantiochytrium* sp. strain TC 20; bars represent standard error (*n* = 2). TAG = Triacylglycerol, PL = Phospholipid, FFA = Free fatty acid, DAG = Diacylglycerol, ST = Sterol, HC = Hydrocarbon (includes Wax ester).

Fatty acid profiles for the thraustochytrid oil derived from *Aurantiochytrium* sp. strain TC 20 were analysed by GC and GC-MS and the main PUFA was DHA 22:6ω3, which was detected at up to 40% of the total FA (Figure 1). The relatively simple fatty acid profile of this strain, having both saturated fatty acids (47% 16:0) and DHA (39%) as the two major constituents is shown in Figure 1. Furthermore, the lipid class profile of *Aurantiochytrium* sp. strain TC 20 also contained a similarly simple profile with 93% triacylglycerol and 3% phospholipid (Figure 2).

### Thraustochytrid and Marine Oils—^13^C Nuclear Magnetic Resonance Spectroscopy

**Figure 3:**
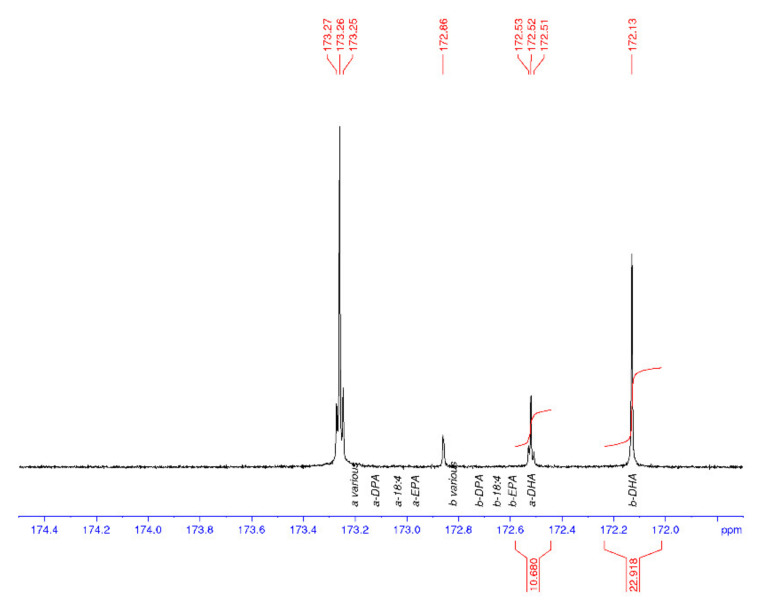
^13^C NMR spectrum of the carbonyl (C1) region of thraustochytrid *Aurantiochytrium* sp. strain TC 20 oil, with DHA enriched in the *sn*-2 position (b). a = *sn*-1,3 and b = *sn*-2.

**Figure 4:**
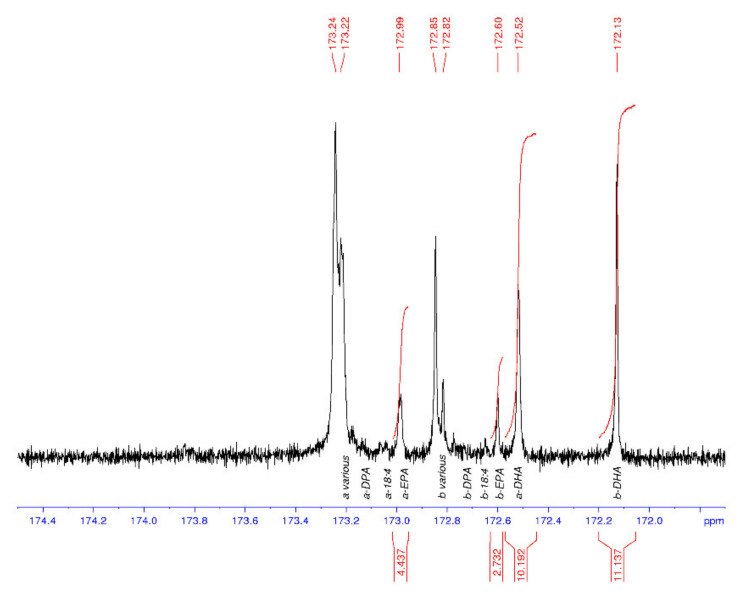
^13^C NMR spectrum of the carbonyl (C1) region of tuna oil with DHA oil enriched in the *sn*-2 position (b). EPA is also enriched at the *sn*-2 position (b). a = *sn*-1,3 and b = *sn*-2.

**Figure 5:**
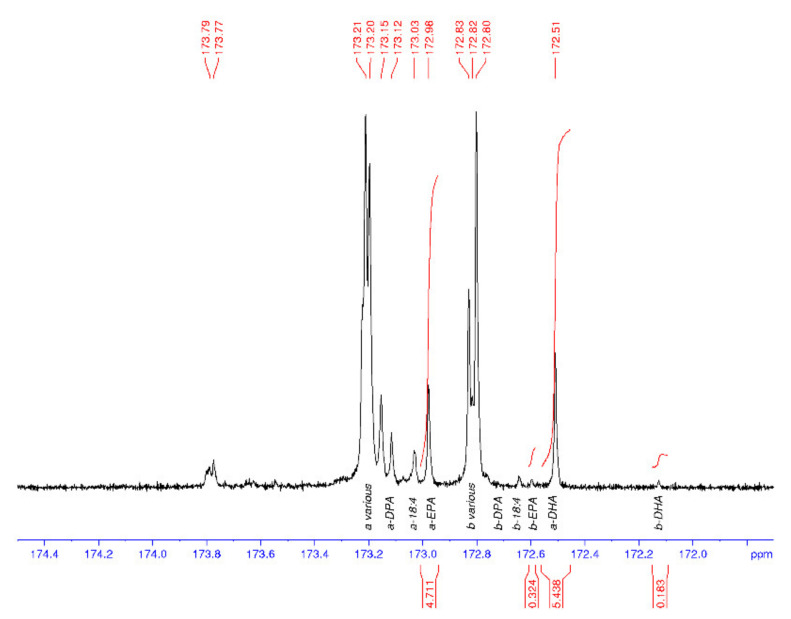
^13^C NMR spectrum of the carbonyl (C1) region of seal oil with DHA, DPA, and EPA enriched at the *sn*−1 and *sn*-3 position (a). a = *sn*-1,3 and b = *sn*-2.

^13^C NMR spectroscopy was used to characterise the regiospecificity of omega-3 LC-PUFA in the thraustochytrid strain TC20 and other marine oils, specifically within the triacylglycerols. The DHA present in the TC 20 TAG fraction was determined to be concentrated in the *sn*-2 position (Figure 3), similar to that found in salmon and tuna oil (Figure 4), and differing to seal oil containing largely *sn*-1,3 LC-PUFA (Figure 5).

Table 1 shows the average and standard deviations (SD) for the a to b ratio, where *sn*-1,3 is a and *sn*-2 is b, and the percentage of DHA in the samples is calculated from the carbonyl resonances. The integrals of the respective signals are provided, relative to the whole C=O region integral being set to 100.0 for each spectrum. The percent DHA values are used to provide quality control for the NMR analysis, in that, if the DHA peaks are incorrectly assigned, then the %DHA would be significantly different to the GC values. The percent DHA of the thraustochytrid oil was determined to be 39% of TFA based on GC analysis, whereas by using NMR it is calculated to be 35%.

The tuna oil DHA% composition values are 25% (GC) and 22% (NMR), which are in general agreement. The GC values are considered to be a better indication of the %TFA composition than the NMR data. Additionally, the NMR values will probably appear low as the range across all peaks including the baseline have been integrated and compared with the DHA peaks, rather than using the sum of all integrated peaks as the divisor. Each NMR data set was processed three times, each on a different day, to provide the calculated values. The seal oil spectrum had an exceptionally small peak for the b-DHA (signal-to-noise of approx. 2), leading to a highly variable peak area integral value and the high SD (a/b). 

**Figure 6:**
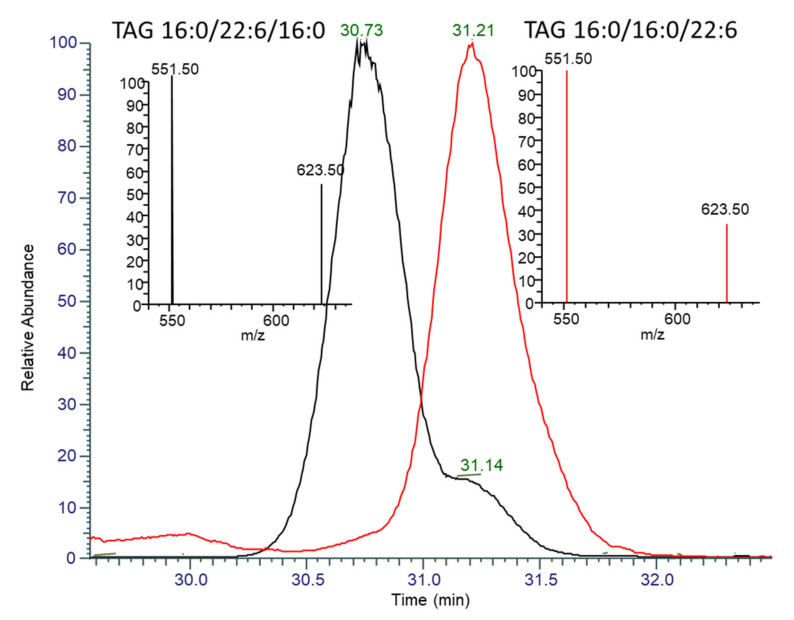
Overlaid extracted ion chromatograms and MS/MS spectra of TAG 54:6 (*m*/*z* 896.77) for Table 2. (black) and seal oil (red). The peak at 30.73 min in TC 20 has a MS/MS spectra (inset left, black) indicative of having DHA in the *sn*-2 position, with the neutral loss of 16:0 at 623.5 *m*/*z* being greater than half the abundance of the neutral loss of DHA at 551.5 *m*/*z*. The peak at 31.2 min in both seal oil and TC 20 has a MS/MS spectra (inset right, red-seal oil) with a neutral loss of 16:0 less than 40% of DHA, indicating preferential fragmentation of *sn*-1,3 DHA.

The LC-MS analysis provided a qualitative description of the lipid species present in the thraustochytrid *Aurantiochytrium* sp. strain TC 20 oil. The major DAG species were DAG 38:6 both (16:0/22:6) and (22:6/16:0), DAG 44:12 (22:6/22:6), DAG 38:5 (16:0_22:5), and DAG 44:11 (22:5_22:6), where “_” denotes that the *sn* position cannot be determined. The major phospholipid species were PC 38:6 (16:0_22:6), PC 44:12 (22:6/22:6), and PC 44:11 (22:5_22:6). The predominant TAG species observed in the thraustochytrid oil by LC-MS/MS was TAG 54:6, which accounts for approximately 35% of the TAG species. The use of a C_30_ column enabled the separation of TAG structural isomers, and also enabled some separation of *sn*-isomers. The positional distribution of the thraustochytrid TAG 54:6 was confirmed to be mainly 16:0/22:6/16:0 through: The separation of the isomers, the confirmed MS/MS fragmentation pattern, and by comparison of the ratio to seal oil TG 54:6 (16:0/16:0/22:6), which eluted at 31.2 min (Figure 6). Whilst the collisional induced dissociation of TAG species with LC-PUFA result in the preferential loss of the LC-PUFA over shorter chain saturated fatty acids, the distribution on the *sn*-2 position also influences the fragmentation of the acyl chains as a second order event. The use of these characteristics and comparison with seal oil, which has a low percent of *sn*-2 DHA, has enabled the characterisation of the TAG species that account for 80% of the thraustochytrid oil (Table 2). Six of these lipid species have *sn*-2 DHA and make up 67% of the total TAG composition.

TAG with LC-PUFA at the *sn*-2 position in intracellular organelles such as lipid droplets are seen by some authors as the most effective and efficient way to store LC-PUFA in lipid and then to place LC-PUFA into membrane phospholipid compared to LC-PUFA occurring at the *sn*-1/3 position [13,14]. The dietary *sn*-2 position of a particular fatty acid, is preferentially incorporated into tissue phospholipid using a rat model [15]. In another rat model study, it was also reported that the EPA and DHA were predominantly at the *sn*-2 position and were more readily absorbed than when present at the *sn*-1 and *sn*-3 positions [16]. In contrast, DHA content was reported to be similar in both seal oil and fish oil-fed rats, even though seal oil DHA is mainly at a *sn*-1 and *sn*-3 position compared to fish oils with DHA at a *sn*-2 position, respectively [17]. The different *sn* positions of the omega-3 LC-PUFA could potentially be affecting the metabolic processes. LC-PUFA, when esterified in the *sn*-2 position of TAG, are thought to be more nutritionally efficacious. DHA concentrated in the *sn*-2 TAG position is observed in other single cell-derived oils, such as 42.6% reported in *Crypthecodinium cohnii* [18]. It is consistent with a report that 60.9% of the DHA was located at the *sn*-2 position in *Schizochytrium* sp. with fatty acid analysis being performed after pancreatic lipase hydrolysis [19]. 

A further comparison to be undertaken was that of the regiospecificity of the thraustochytrid-derived oil, and that of new oils obtained in recent years from genetically engineered plants producing oil seeds including *Arabidopsis*, *Camelina*, and canola [20,21,22]. The thraustochytrid *Aurantiochytrium* sp. strain TC 20 oil contains DHA preferentially located at the *sn*-2 position as noted above. The thraustochytrid oil shares this feature with the more standard fish oils, as confirmed in our study. In contrast, the new plant seed-derived oils all contain DHA largely at *sn*-1,3 positions [23]. This observed difference in regiospecificity between single cell oils and new oil seed-derived products is a key feature distinguishing these two novel alternative sources of long-chain omega-3 oils.

In conclusion, we have presented a ^13^ C NMR application that permits regiospecificity characterisation of thraustochytrid sourced TAG containing oil in a comprehensive manner. These results were complemented by further oil characterisation using LC-MS/MS to determine the lipid species. The study determined that the TAG DHA is enriched at the *sn*-2 position in the Australian thraustochytrid strain TC 20, with TAG (16:0/22:6/16:0) the main species present. 

## 3. Materials and Methods

Thraustochytrid *Aurantiochytrium* sp. strain TC 20 used in this study is deposited in the Australian National Algae Culture Collection (http://www.csiro.au/ANACC). Strain isolation information, medium preparation, and culturing conditions have been reported previously [6,24]. Samples (100 mg of freeze dried biomass) were extracted quantitatively by the modified dichloromethane (DCM)-methanol (MeOH)-water Bligh and Dyer (1959) method [25]. After phase separation, the lipids were recovered in the lower DCM layer and a second extraction performed to maximise lipid recovery. Solvents were removed in vacuo. Lipid recovery was determined gravimetrically. An aliquot of the total lipid was made up to a known volume for TLC-FID analysis. Marine oils analysed were supplied by commercial sources: tuna oil, Pharmamark Nutrition Pty Ltd (Brisbane, Australia); seal oil, fish, salmon, Solgar and Neuromins oils were purchased as supplements from retail outlets.

### 3.1. Lipid Class Composition

Aliquots of each of the diluted oils were analysed using an Iatroscan MK V TLC-FID (Iatron Laboratories, Tokyo, Japan) analyser to determine the abundance of individual lipid classes. Samples were applied to silica gel SIII chromarods (5-μm particle size) using 1-μL disposable micro pipettes. The chromarods were developed in a glass tank lined with pre-extracted filter paper. The solvent system used for the lipid separation was hexane-diethyl ether-acetic acid (60:17:0.2 v/v/v), a mobile phase providing good resolution between non-polar compounds such as wax ester, triacylglycerol, and free fatty acid. After development (30 min), the chromarods were oven dried (8 min) and analysed immediately to minimise the adsorption of atmospheric contaminants. Data presented are for qualitative percentages of individual lipid classes. Iatroscan results have been previously shown to be reproducible to ±10% or better [26].

### 3.2. Methylation and Analysis of Fatty Acid Methyl Esters (FAME)

An aliquot of the extracted lipids were transesterified with methanol/dichloromethane/HCl (10:1:1 v/v/v) to convert fatty acids from their complex lipids into FAME, as described previously [27]. Individual fatty acids are expressed as a percentage of the total fatty acids (TFA). Gas chromatography (GC) was used to quantify the fatty acids and was performed on an Agilent Technologies 7890A GC (Palo Alto, CA, USA) equipped with a nonpolar Equity-1™ fused silica capillary column (15 m × 0.1 mm i.d., 0.1-mm film thickness), flame ionisation detector, and split/splitless injector. Samples were injected in splitless mode at an oven temperature of 120 °C, and after injection, the oven temperature was increased to 270 °C at 10 °C/min and then to 310 °C at 5 °C/min. Peaks were quantified with Agilent Technologies ChemStation software (Palo Alto, CA, USA).

GC–mass spectrometry (GC-MS) analysis of FAME was performed to confirm individual component identifications and was carried out on a ThermoScientific 1310 GC coupled with a TSQ triple quadruple. Samples were injected using a Tripleplus RSH auto sampler with analyses performed using a non-polar HP-5 Ultra 2 bonded-phase columns (50 m × 0.32 mm i.d. × 0.17 µm film thickness). The HP-5 column was of a similar polarity to the column used for the GC analyses. The initial oven temperature of 45 °C was held for 1 min, followed by an increase in temperature of 30 °C per minute to 140 °C, then at 3 °C per minute to 310 °C, where it was held for 12 min. Helium (He) was used as the carrier gas. The operating conditions of the GC-MS were: Electron impact energy 70 eV; emission current 250 µamp, transfer line 310 °C; source temperature 240 °C; scan rate 0.8 scan/sec; and mass range of *m*/*z* 40–650. Thermo Scientific XcaliburTM software (Waltham, MA, USA) was used to acquire and process mass spectra.

### 3.3. ^13^C Nuclear Magnetic Resonance Spectroscopy (^13^C NMR) Analysis

The samples were made up to a total volume of 0.6 ml with deuterio chloroform containing *ca*.25 mM tris(acetylacetonate) chromium (III) as a relaxation agent. Quantitative ^13^C NMR spectra were acquired on a Bruker BioSpin Av500 NMR spectrometer equipped with a 5-mm ^1^H-^13^C-^15^N inverse triple resonance probe with *z* gradient operating at 125.8 MHz for ^13^C. The samples were maintained at 25 °C during acquisition. A total of 128 k data points were collected over a spectral width of 26.3 kHz summed over 12–64 k scans.

Inverse gated, bilevel ^1^H decoupling was employed with an acquisition time of 2.49 s and a recycle delay of 2.5 s. The data were processed in Bruker BioSpin TopSpin v3.1 over 128 k data points using a Gaussian multiplication with a Gaussian position factor of 0.12 and a line broadening of 0.15 Hz; a 5th order polynomial baseline correction was applied to each spectrum. Spectra were referenced to the peak arising from C_1_ of 22:6ω3 in the *sn*-2 position of triacylglycerols at 172.13 ppm [28] and the signals were assigned using the published assignments of Aursand et al. [29]. Each of the raw data were processed in a similar fashion in triplicate and the average and standard deviations calculated.

### 3.4. Liquid Chromatography Mass Spectrometry Analysis

Oils were diluted 1:10,000 in a 50:50 mixture of butanol and methanol with 0.05% butylated hydroxy toluene The samples were separated on a C_30_ Acclaim (2.1 × 250 mm, 3 µm) column (ThermoFisher, Scoresby Victoria), based on the manufacturer’s instructions with modifications to optimise for TAG *sn*-isomer separation. The column was held at 20 °C, over a gradient comprised of solvents: A, 30% methanol, 30% acetonitrile, 0.1% formic acid, and 10 mM ammonium formate, with water, as well as B, 100% isopropanol. The gradient commenced at 50% B and ramped to 80% at 10 min, followed by a gradual increase to 85% at 25 min, held until 35 min and a final increase to 90% at 50 min before re-equilibration to 50%.

The samples were analysed on a ThermoFisher Orbitrap Fusion Tribid mass spectrometer using a heated electrospray ionisation source (H-ESI). The H-ESI conditions were a positive ion spray voltage at 3500 V, nitrogen was used as the desolation gas for the sheath, auxiliary, and sweep gas at 35, 5, and 1 arbitrary units, respectively. The ion transfer tube and vaporiser temperature were both set to 300 °C. An accurate mass of each lipid species was measured using the orbitrap mass analyser at a resolution of 60,000 in the scan range of 250–1500 *m*/*z* in positive ion mode, the AGC target was set at 40,000 with a maximum injection time of 50 milli sec, and RF lens set at 45%. Data dependent acquisition was conducted on masses with an intensity threshold above 20,000 counts, excluded for 6 s after MS/MS were obtained. The data dependent MS/MS spectra were isolated in the quadrupole with an isolation window of 0.7 *m*/*z*, fragmented in the HCD collision cell at 30% collision energy, and data obtained in the orbitrap at 15 K resolution.

LC-MS/MS data were analysed using LipidSearch 4.2 (ThermoFisher Scientific, Scoresby, Victoria, Australia) for lipid species annotation and retention time. Masses and fragmentation data were imported into TraceFinder (ThermoFisher) for peak area quantification. Further qualitative analysis was conducted using Freestyle (ThermoFisher).

## Figures and Tables

**Table 1:** The average and standard deviations (SD) for the a to b ratio and the percentage of DHA in algal and marine oil samples calculated from the carbonyl resonances of the ^13^C NMR analysis (*n* = 3).

	%DHA	SD	a-DHA: *sn*-1+3	b-DHA: *sn*-2	a/b *sn*-1+3 /*sn*-2	SD	a-EPA: *sn*-1+3	b-EPA: *sn*-2	a/b *sn*-1+3/ *sn*-2
Algal oils									
*Aurantiochytrium* sp. strain TC 20 oil	35.2	1.5	10.68	22.93	0.47	0.03	0	0	
Neuromins Algal DHA oil	36.2	0.4	16.66	19.19	0.87	0.003	0	0	
Solgar Algal DHA oil	17.8	0.5	4.56	4.25	1.07	0.001	0	0	
Marine oils									
Healthy Care Fish oil	10.1	0.2	3.80	6.04	0.63	0.01	12.99	3.26	3.99
Tuna oil	21.6	0.3	10.19	11.14	0.92	0.01	4.44	2.73	1.62
Swisse Salmon oil	6.9	0.3	2.02	4.86	0.42		5.07	3.50	1.45
Seal oil	6.0	0.3	5.44	0.18	29.70		4.71	0.32	14.55

**Table 2:** Lipid composition of TAG species of thraustochytrid *Aurantiochytrium* sp. strain TC 20 oil, as analysed by LC-MS/MS.

Sum Composition	Lipid Species	*m*/*z*	TAG%
TAG(54:6)	TAG (16:0/22:6/16:0)	896.77	34.4
TAG(60:12)	TAG (16:0/22:6/22:6)	968.77	16.6
TAG(52:6)	TAG (16:0/22:6/14:0)	868.74	7.3
TAG(54:5)	TAG (16:0/22:5/16:0)	898.79	6.3
TAG(60:11)	TAG (16:0/22:6/22:5) and TAG (16:0/22:5/22:6)	970.79	4.8
TAG(48:0)	TAG (16:0/16:0/16:0)	824.77	3.4
TAG(56:6)	TAG (16:0/22:6/18:0)	924.8	2.6
TAG(46:0)	TAG (16:0/16:0/14:0)	796.74	2.1
TAG(52:5)	TAG (16:0/22:5/14:0)	870.75	1.5
TAG(66:18)	TAG (22:6/22:6/22:6)	1040.77	1.5
